# Biology of PEST‐Containing Nuclear Protein: A Potential Molecular Target for Cancer Research

**DOI:** 10.3389/fonc.2022.784597

**Published:** 2022-02-04

**Authors:** Nazeer Hussain Khan, Hao-Jie Chen, Yuanyuan Fan, Muhammad Surfaraz, MD.Faysal Ahammad, Yang-Zhe Qin, Muhammad Shahid, Razia Virk, Enshe Jiang, Dong-Dong Wu, Xin-Ying Ji

**Affiliations:** ^1^ Henan International Joint Laboratory for Nuclear Protein Regulation, School of Basic Medical Sciences, Henan University, Kaifeng, China; ^2^ School of Life Sciences, Henan University, Kaifeng, China; ^3^ Faculty of Pharmacy, The University of Lahore, Lahore, Pakistan; ^4^ Key Laboratory of Natural Medicine and Immune Engineering, School of Medicine, Henan University, Kaifeng, China; ^5^ Department of Biological Sciences and Biotechnology, Faculty of Science and Technology, Universiti Kebangsaan Malaysia, Bangi, Malaysia; ^6^ Department of Bio-Sciences, University Wah, Rawalpindi, Pakistan; ^7^ Institute of Nursing and Health, Henan University, Kaifeng, China; ^8^ School of Stomatology, Henan University, Kaifeng, China; ^9^ Kaifeng Key Laboratory of Infection and Biological Safety, Henan University College of Medicine, Kaifeng, China

**Keywords:** cancer, PEST-sequence, nuclear proteins, molecular research target, PCNP, therapeutics

## Abstract

PEST-containing nuclear protein (PCNP), a novel nuclear protein, is involved in vital cellular processes like cell proliferation and mediates tumorigenesis. PCNP is a short-living, small nuclear protein of only 178 amino acids with two remarkable PEST sequences that are rich in proline (P), glutamic acid (E), serine (S), and threonine (T). The current understanding of PCNP reveals that PCNP has the ability to interact with cell cycle regulatory proteins; tumor suppressors (p53 and pRB), and promoters (cyclin E and cyclin D) to determine the fate of tissues to facilitate the process of either apoptosis or cell proliferation. In many preclinical studies, it has been evaluated that PCNP expression has associations with the development and progression of various cancers like neuroblastoma, lung adenocarcinoma, and ovarian cancer. Based on these depicted novel roles of PCNP in cell cycleregulation and of PCNP in tumorigenesis, it is logical to consider PCNP as a potential molecular target for cancer research. The aim of the current communication is to present an update on PCNP research and discussion on the potential role of PCNP in cancer development with challenges and opportunities perspectives. Considering the available evidence as a baseline for our statement, we anticipate that in the future, new research insights will strengthen the aim to develop PCNP-based diagnostic and therapeutic approaches that will move the PCNP from the laboratory to the cancer clinic.

## Introduction

To maintain normal continuity of cell life, nuclear proteins (NPs) have central importance in regulating vital innate mechanisms. Molecular and biochemical evidences have attested to the notion that NPs with amazing features play important roles to regulate the cell division and stem cell generation, condense and repair the genomic contents, organize the nuclear metabolism, and maintain the architecture of cell machinery with the process of ubiquitination (1–6). Based on their nature and functions, a family of NPs, rich in proline (P), glutamic acid (E), serine (S), and threonine (T) sequences, known as PEST-proteins (PEST-NPs), is widely distributed and involved in various cellular functions. This class of PEST-NPs is known as the guardian of the cell and is believed to play roles in the ubiquitin–proteasome pathway, glycosylation of nuclear pores, and the hexosamine biosynthetic pathways (7, 8). Furthermore, these proteins are considered active players in cellular processes like cell cycle regulation, cell nutrient uptake and cellular metabolism regulation, nucleocytoplasmic transport and regulating cyclic nucleotide signaling pathways (9–12). Some of the PEST-NPs are primarily involved in regulating cancer metabolism by interfering with key signaling pathways and cancer-immune mechanisms *via* apoptosis and autophagy (7). In particular, there is a growing volume of evidence about PEST-NPs PEST sequence function as proteolytic signals to target the degradation of different proteins *via* the proteasome or by calpain proteolysis pathways (8, 13, 14).

In 2002, through database mining, Mori and his co-workers discovered a novel PEST-containing protein, named PCNP, localized in the nucleus. Rich in these PEST sequences, PCNP is a novel short-living NP and regulates many cellular activities in the form of a transcriptional factor or acting as cell cycle regulatory proteins (15).

Through the proteomic studies databases and *in vitro*–*in vivo* experiments, PCNP expression has been confirmed in several cancer cells, including HepG2 hepatoma cells, U-937 myeloid leukemia cells, and HT-1080 fibrosarcoma cells (15, 16). Furthermore, studies confirmed the potential mediating role of PCNP in the proliferation, migration, and invasion of human neuroblastoma, lung adenocarcinoma cells, and ovarian cancer cells. Findings demonstrate that PCNP is involved in disrupting key cellular signaling cascades like PI3K/AKT/mTOR and MAPK, which lead to the development of different cancers (17, 18). Results of a recent novel study depicted that PCNP is involved in promoting ovarian cancer progression through activating the Wnt/β-catenin signaling pathway and epithelial–mesenchymal transition (EMT) (19).

Herein, we have summarized the available literature on the biology of PCNP and its potential role as a molecular target in the different cellular activities through which it takes part in different cancer developments. Considering the available evidence of a candidate protein as a potential target in cancer research, we anticipate that in-depth further exploration into elucidating the precise associations of PCNP and mechanisms of cancer progression holds great promise for PCNP use as a diagnostic and therapeutic target for different cancers.

## Ubiquitination of PCNP

**Figure 1:**
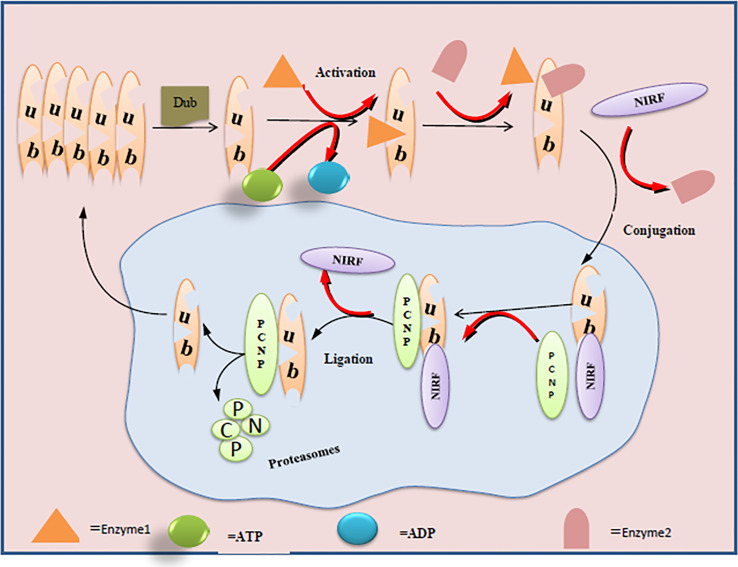
Ubiquitination of PCNP by NIRF in the nuclear cell: ligase enzymes (E1, E2) facilitate the conjugation and ligation of NIRF with PCNP. C-terminus of NIRF performs the ubiquitin ligase function and acts on the PCNP as substrate and mediates the proteasome activity.

Inside a cell, ubiquitination is the most dynamic and highly controlled biological phenomenon to mark proteins for degradation, control their activity, alter their localization, and reinforce or hinder their protein interactions (20, 21). These crucial phenomena regulate protein functions, happen *via* special types of protease, and determine cell survival, differentiation, and other physiological processes (22, 23). Owing to its PEST sequences, PCNP is likely to be very prone to the machinery of ubiquitination in cells (15). Through the mammalian two-hybrid system, GST pull-down assay, and nuclear co-localization studies, it has been evaluated that PCNP shows interaction with ubiquitin-like Np95/ICBP90‐like RING finger protein (UHRF2/NIRF) (16). NIRF is a ubiquitin multi-domain E3 ligase that plays a very important role in maintaining DNA methylation, regulating cell cycle *via* p53, and DNA damage (24–27). Later on, in a follow-up study through co-immunoprecipitation and Western blotting analysis, it has been found that NIRF is a potential ubiquitin ligase and is capable of ubiquitinating the PCNP (16). Going through these *in vivo* and *in vitro* validations of PCNP ubiquitination through NIRF highlights the importance of NIRF as the target to determine the potential role of PCNP in different cancers. In addition, these findings also open a new avenue to investigate the mutual roles of NIRF and PCNP in different signaling pathways concerned with cell cycle regulation and genome stability. **Figure 1** shows the mechanisms of PCNP ubiquitination by NIRF.

## Transcriptional Pattern of PCNP With Other Proteins

The help of new high-throughput technologies and advanced levels of bioinformatics tools has enabled the scientists to elucidate the functions of the large fraction of genes in the genome whose functions are currently unknown and help to discover how different genes interact to perform biological functions. Expression patterns of a gene determine the functionality of the protein encoded. Published literature reveals that genes that encode the proteins participating in the same pathway or part of the same protein complex are often co-regulated and exhibit the same expression patterns (28–30); however, co-regulation does not necessarily imply that genes are functionally related (28).

### Co-Expression of PCNP and PPP1CC

Protein phosphatase 1 (PP1) is a major eukaryotic protein serine/threonine phosphatase that regulates an enormous variety of cellular functions through the interaction of its catalytic subunit (PP1c). It is an indispensable component in the process of cell division and apoptosis and participates in the regulation of glycogen metabolism, muscle contraction, and protein synthesis (31–33). Employing the weighted gene co-expression network analysis (WGCNA) in a large-scale blood gene expression study on amyotrophic lateral sclerosis (ALS) patients, it has been found that PCNP shows a similarity in pattern expression to PPP1CC (34). The findings give insights into developing the association of PCNP function with PPP1CC and demand further exploration of their co-expression and synchronization in functions.

### Co-Expression of PCNP and CGREF1

Cell growth regulator with EF hand domain protein 1 (CGREF1) is involved in mediating cell adhesion by binding to calcium and inhibiting the proliferation of some cell lines (35, 36). It has been evaluated that PCNP shows mRNA transcription level similarity with CGREF1 in the regulation of the hepatoma cell cycle in the zebrafish hepatoma model (37). Considering the importance and evidence of expression pattering in genes, this mRNA transcription similarity of PCNP with a protein involved in cell adhesion and proliferation predicts the PCNP role in cancer cell metastasis.

### Co-Expression of PCNP and SRP9

In 2016, de Jong and his team investigated attention-deficit hyperactivity disorder (ADHD) and major depressive disorder (MDD). In whole genome blood gene expression analysis and genetic risk scores of 318 individuals, they found that PCNP shows co-expression with Signal recognition particle 9-kDa protein (SRP9) (38).

SRP9 plays an important role in directing secretory proteins to the rough endoplasmic reticulum. SRP9, together with the Alu part of SRP14 and SRP RNA, constitutes the extended inhibition domain of SRP. The complex of SRP9 and SRP14 is necessary for the binding of SRP RNA (39, 40). Without any doubt, this co-expression of PCNP with SRP9 holds the promise of important association and PCNP functions on further exploration.

### Co-Expression of PCNP and DPM1

Dolichol-phosphate mannosyltransferase subunit 1 (DPM1) is a mannose donor in various glycosylation reactions and a catalytic subunit of the polyol-mannose phosphate (DPM) synthesis complex (41–43). As with other proteins like PPP1CC, PCNP have the co-expression pattern with DPM1 at the post-translational modification level in patients with ALS (43). DPM1 and PCNP co-expression could be considered, as synchronization of PCNP functions with DPM1 and demands in-depth exploration.

## PCNP Kinship With Other Proteins

As mentioned earlier, the expression patterns of a gene determine the functionality of the protein encoded. Transcription patterning analysis of a PCNP showed that it is co-expressed with MARCH7, BMI1, TMEM123, TRAM1, and PSMC6. The function of MARCH7 includes regulating DNA damage through the p53 gene, regulating cell migration, invasion, and autophagy induced by TGF-β (44, 45). BMI1 proto-oncogene, polycomb ring finger (BMI1) can form a PCG–PRC1 complex, which plays a role through chromatin remodeling and histone modification, and can mediate the ubiquitination of histones, resulting in hereditary changes in the expression rate of chromatin (46, 47). Moreover, co-expression of MARCH7, BMI1, and PCNP is associated with ALS, but the correlation is low (34). Transmembrane protein 123 (TMEM123) can cause cancer cell swelling, cancer cell organelle swelling, vacuolization, and increased membrane permeability, thus participating in the death of tumor cells (47). Translocation associated membrane protein 1 (TRAM1) stimulates secretory proteins through the endoplasmic reticulum membrane and promotes the translocation of endoplasmic reticulum membrane proteins (48). Proteasome 26S subunit (PSMC6), a component of 26S proteasome, is a multi-protein complex that participates in ubiquitin recognition of proteasome and plays a key role in maintaining protein dynamic balance by removing misfolded or damaged proteins that may damage cell function, and by removing proteins with functions that are no longer needed (49). However, PCNP and these three proteins are only co-expressed experimentally, and the role of their co-expression has not been reported in the literature.

## Multifaceted Behavior PEST-Containing Nuclear Protein

PEST sequence proteins are considered guardians of the cell and perform vital physiological functions. These proteins execute the process of the ubiquitin–proteasome pathway, glycosylation of nuclear pores, and the hexosamine biosynthetic pathway (8). In initial research on PEST-containing protein PCNP, it has been found that, on the one hand, PCNP is ubiquitinated by NIRF as a tumor suppressor cell regulatory protein in neuroblastoma like p53 and pRB (17). On the other hand, it acts as a tumor-promoting cell regulatory protein in lung adenocarcinoma like cyclin D and cyclin E (18). Furthermore, overexpressed PCNP mediates apoptosis *via* mitochondrial-mediated pathway, which is evident with a higher apoptotic index and higher expressions of cleaved caspases 3, 8, and 9 as compared with control in Western blotting analysis. However, the exact mechanism of the role of PCNP in mediating cancer cell death is not clear. It has been supposed that PCNP may bind to the DNA directly inside the nucleus and start apoptosis *via* specialized proteins or after being localized in the cytosol and mediating the mitochondrial-dependent apoptotic pathway (7).

## PCNP as Potential Therapeutic Target for Different Cancers

### PCNP Promotes the Progression of Ovarian Cancer

Owing to its involvement in the regulation of cell proliferation, apoptosis, and EMT, the Wnt signal pathway has been considered an important pathway to investigate the development and progression of tumors (50, 51).

**Figure 2:**
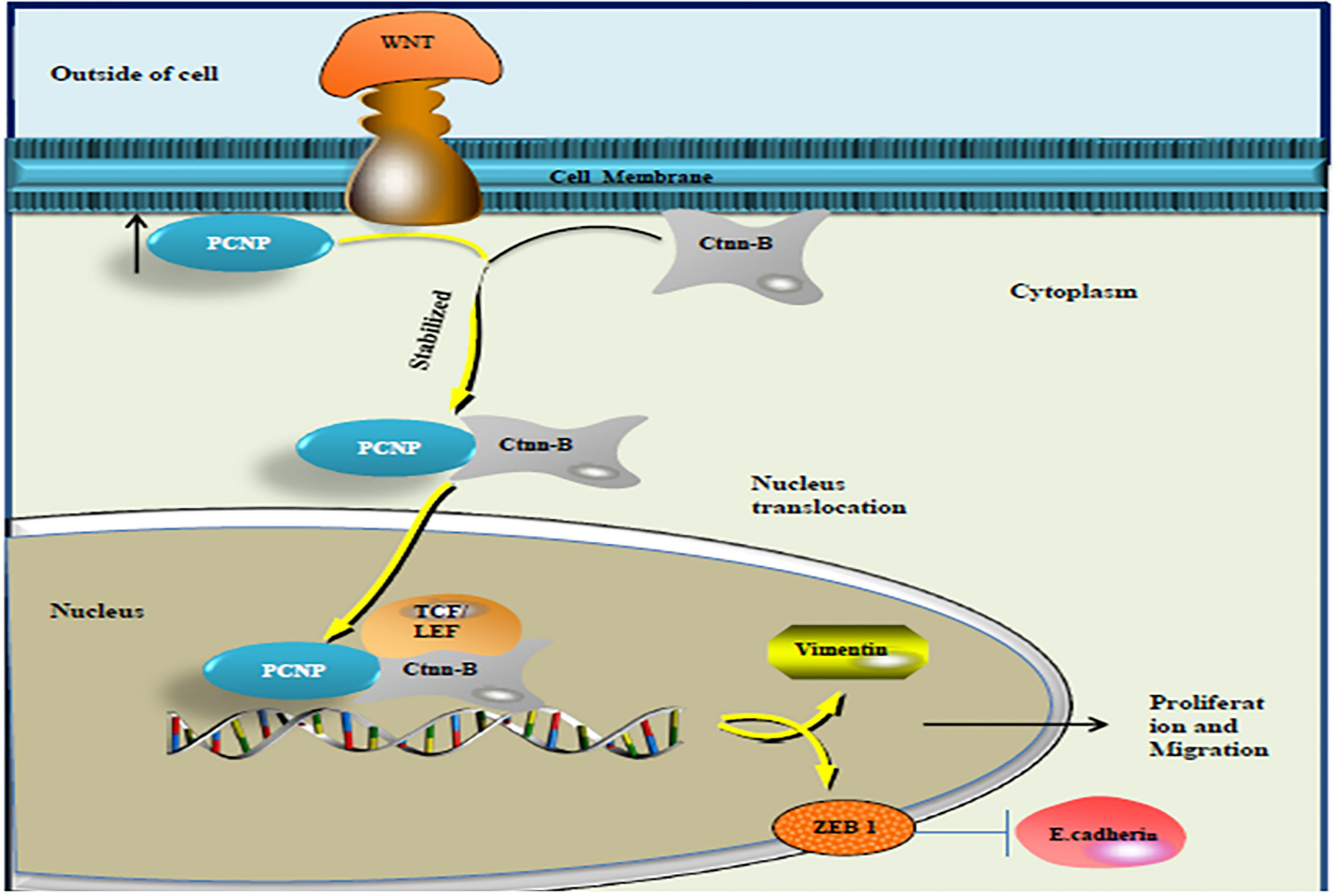
PCNP binding with β catenin: after binding with β catenin, PCNP becomes stabilized and accelerates the expression of β catenin in the nucleus, which further activates the Wnt signaling pathway and promotes ovarian cancer progression.

In particular, it has been found that any sort of aberration in Wnt pathway proteins imposes a significant impact on the ovarian tissues and may become worse in the form of ovarian cancer (52). Dong et al. found a novel association of PCNP with the Wnt pathway. Their results indicate that PCNP is overexpressed in both ovarian cancer tissues and cells more than those in para-cancerous tissues and ovarian epithelial cells (IOSE80), respectively. Through *in vitro* and *in vivo* experiments on ovarian cancer cells (SK-OV-3 and A2780) and xenografted nude mice models, it has been found that high expression of PCNP promotes the growth, migration, and invasion of ovarian cancer cells and inhibits apoptosis. They further evaluated that PCNP binds to β-catenin and promotes β-catenin nuclear translocation and then activates the Wnt/β-catenin signal pathway (19). The findings suggest that PCNP may be a regulatory protein upstream of the Wnt pathway involved in the occurrence and development of ovarian cancer and could act as a novel target for treating ovarian cancer. **Figure 2** illustrates the pathway of PCNP and catenin β in ovarian cancer.

### Overexpressed PCNP Promotes Adenocarcinoma

Lung cancer is the leading cause of cancer-related mortality worldwide. A study published in *Oncogenesis* observed that PCNP is overexpressed in the human lung adenocarcinoma tissues more than in the corresponding adjacent non-tumor tissues. Overexpression of PCNP promoted the proliferation, migration, and invasion of adenocarcinoma cells, while knockdown of PCNP exhibited the opposite effects. The authors further investigated that overexpressed PCNP mediates the upregulation of the protein expression of p-STAT3 and p-STAT5 and decreases apoptosis. Moreover, compared with the blank control group, it has also been found that in the subcutaneous tumor of nude mice established by A549 and H1299 cells, the growth of tumor cells in the PCNP overexpression group was significantly faster (volume and weight increased, and the tumor doubling time was shortened), and tumor microvessel density (TMD) was also increased (18). These results exploit clearly that PCNP could be considered as a promising biomarker for diagnosis and prognosis in patients with lung adenocarcinoma. Furthermore, designing a potent PCNP inhibitor strategy could be adopted for the treatment of lung adenocarcinoma.

### Upregulated PCNP Inhibits Neuroblastoma

Novel findings on the expression association of PCNP with the development and progression of various cancers have made the PCNP a flashpoint of oncology research and urges the biologists for further exploration.

Recently, PCNP gene-expression (downregulation and upregulation) association with the progression of human neuroblastoma was evaluated by using the SH-SY5Y and SK-N-SH cell lines. Findings show that compared with the normal control group, the high expression of PCNP decreased the growth, migration, and invasion of human neuroblastoma through MAPK and PI3K/AKT/mTOR signaling pathways and promoted the apoptosis of neuroblastoma. Opposite results were observed by silencing the PCNP gene in human neuroblastoma cells. Results were further strengthened by xenograft tumor analysis in animal experiments for both PCNP overexpression and knockdown (17). These novel findings imply that PCNP is a potential therapeutic target for designing drugs to treat patients with neuroblastoma.

## Future Outlook Under Cell-Biology Prospective

The occurrence and development of cancer are the result of the joint action of many factors, not only with the participation of oncogenes such as c-Jun, c-Fos, p53, and other oncogenes, but also by the regulation of signal transduction pathways such as PI3K/Akt, and Wnt (53–55).

Although the current findings have well established the PCNP participation in the regulation of cell cycle, and its role in affecting PI3K/Akt and Wnt signaling pathways in different cancers, however, there are still many dots that are unconnected. For example, there is no study on the direct association of PCNP with signaling molecules and on their impact on cell internal regulation.

In terms of protein–protein interaction, for the normal continuity of cells, each physiological function relies on thousands of important protein–protein interactions (56). PCNP interactions with reported proteins have been partially elucidated, and there are still many problems that have not been solved, such as: a) whether UHRF2 ubiquitination of PCNP leads to its degradation by the proteasome or whether UHRF2 can affect some cancers; and b) whether proteins containing PEST sequence through PCNP can be rapidly degraded and what stabilizes PCNP in tumor cells; c) whether PCNP can be ubiquitinated by UHRF2 and whether MARCH7 can also ubiquitinate PCNP; and d) whether CGREF1 can mediate cell adhesion and inhibit the growth of multiple cell lines and what the effect of co-expression of PCNP and CGREF is on the migration and proliferation of cancer cells. In particular to this study, we also believe that PCNP exploration in system biology perspective will account to gain a better understanding regarding its biology and potential roles in cancers. System-level understanding constructs the theme in cell biology to depict the anatomy and physiology of biomolecules (57). To date, PCNP lacked a three-dimensional (3D) structure on the protein data bank and there was no reported study on docking ability of PCNP with other oncogenic proteins. Through the use of breakthrough advancements in devices and analytical methods with high-throughput measurements in genomic studies, we look forward to examining the structure and cellular dynamics of PCNP functions in biochemical pathways.

## Conclusion Remarks

In conclusion, the current published literature on PCNP represents the critical and clinical roles of PCNP in tumor development, and it highlights the need to develop new understandings of the role and interactions of NPs in regulating the cell cycle. The findings of all preclinical studies on PCNP have established the fact that PCNP is the key regulatory protein in tumorigenesis and has the potential to be considered as a novel molecular target for designing drugs to treat cancer. However, as an attractive molecular target for cancer, it demands exporting the underlying mechanisms of PCNP actions both in mediating apoptosis and in cell proliferation. We anticipate and look forward that in the future, new research insights that will strengthen the aim of developing PCNP-based diagnostic and therapeutic approaches that will move the PCNP from the laboratory to the cancer clinic.

## Author Contributions

NHK, DDW, XYJ, and ESJ participated in the conception and manuscript drafting. NHK, HC, YF, MS, MFA, YQ, MS, RV, did the literature mining and wrote the draft. MS, RV did graphical work and draw the figure to explain pathways of candidate protein. DDW, XYJ, ESJ supervised the study put the intellectual input to the review. All authors read and approved the final manuscript.

## Funding

This work was supported by Henan Provincial Science and Technology Research Project [No. 212102310147], the National Natural Science Foundation of China (Nos. 81900375, 81802718 and 81670088), the Foundation of Science & Technology Department of Henan Province, China (Nos. 202102310480 and 192102310151), and the Training Program for Young Backbone Teachers of Institutions of Higher Learning in Henan Province, China (No. 2020GGJS038).

## Conflict of Interest

The authors declare that the research was conducted in the absence of any commercial or financial relationships that could be construed as a potential conflict of interest.

## Publisher’s Note

All claims expressed in this article are solely those of the authors and do not necessarily represent those of their affiliated organizations, or those of the publisher, the editors and the reviewers. Any product that may be evaluated in this article, or claim that may be made by its manufacturer, is not guaranteed or endorsed by the publisher.

## Abbreviations

PCNP, PEST‐containing nuclear protein; NPs, nuclear proteins; PEST, proline (P), glutamic acid (E), serine (S), threonine (T); EMT, epithelial–mesenchymal transition; MAPK, mitogen-activated protein kinase; mTOR, mammalian target of rapamycin; CGREF1, cell growth regulator with EF hand domain protein 1; SRP9, signal recognition particle 9-kDa protein; DPM1, dolichol-phosphate mannosyltransferase subunit 1; PP1, protein phosphatase 1; NIRF, Np95/ICBP90‐like RING finger protein.
